# Supplementary material for: A naturally occurring mini-alanyl-tRNA synthetase
Source: Commun Biol. 2023 Mar 23;6:314. doi: 10.1038/s42003-023-04699-0 (PMC10036535; doi:10.1038/s42003-023-04699-0)

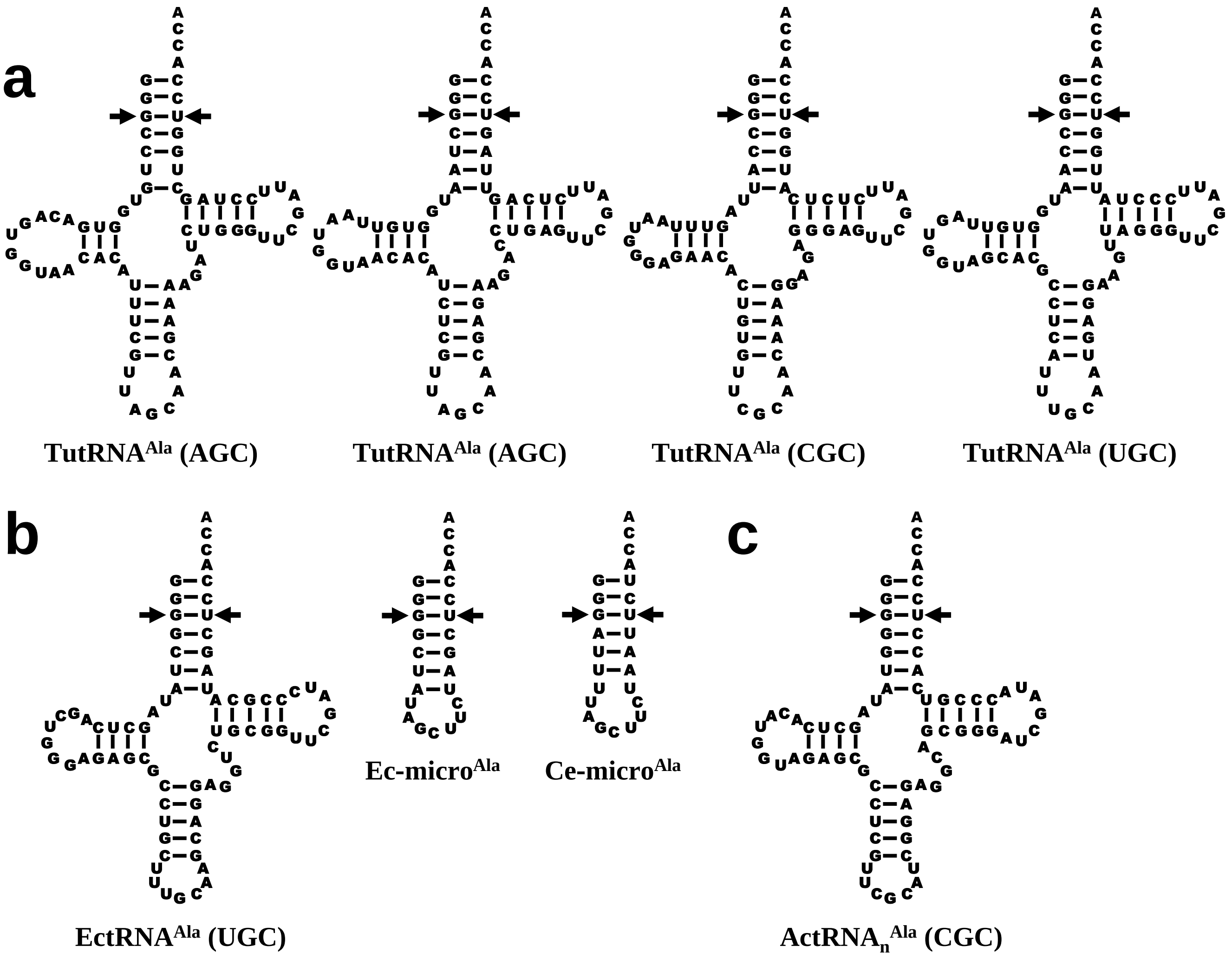

Supplementary Figure 1. Cloverleaf structures of tRNAs. Secondary structures of (a) Tupanvirus $tRNA^{Ala}$ isoacceptors, (b) $EctRNA^{Ala}$, Ec-$micro^{Ala}$, Ce-$micro^{Ala}$, and (c) $ActRNA_n^{Ala}$.

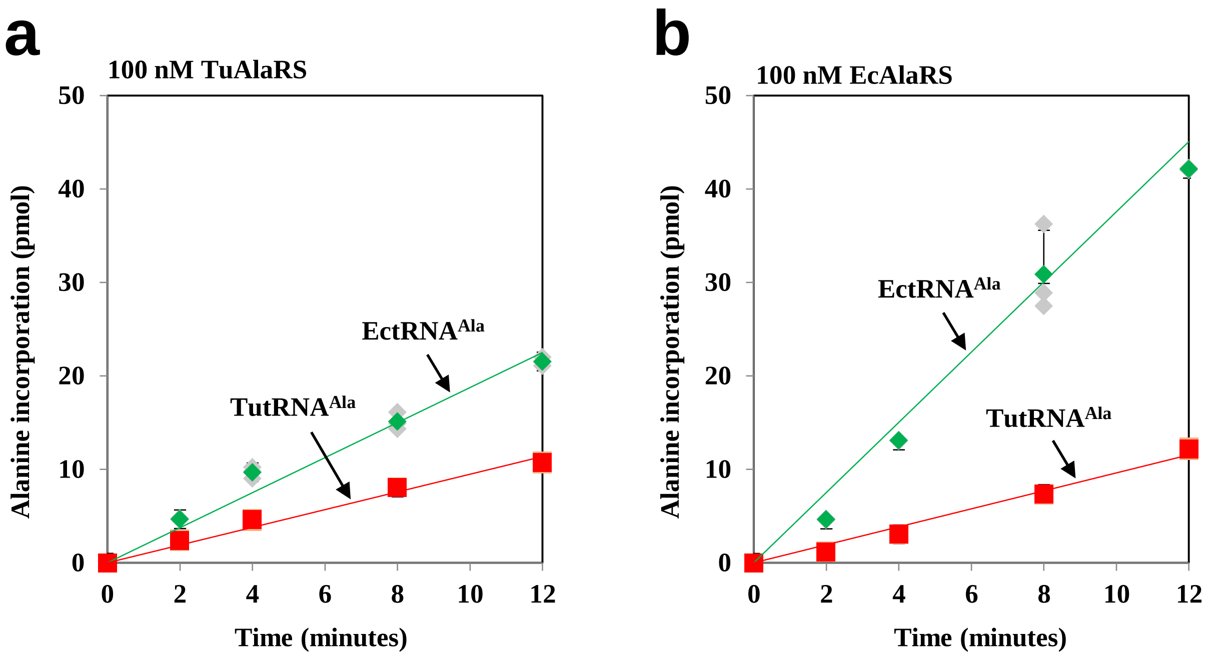

Supplementary Figure 2. Aminoacylation of EctRNA^{Ala} and TutRNA^{Ala} by EcAlaRS and TuAlaRS. Aminoacylation of EctRNA^{Ala} and TutRNA^{Ala} was carried out at 37˚C with (a) TuAlaRS (100 nM) and (b) EcAlaRS (100 nM).

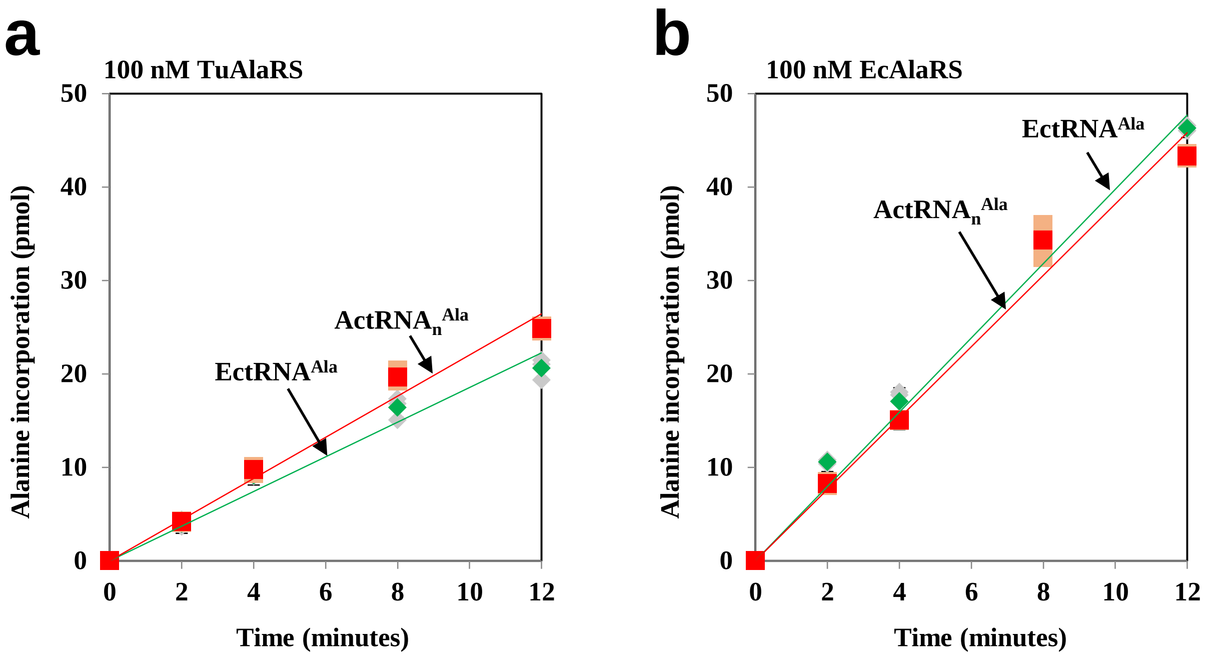

Supplementary Figure 3. Aminoacylation of EctRNA^{Ala} and ActRNA$_n^{Ala}$ by EcAlaRS and TuAlaRS. Aminoacylation of EctRNA^{Ala} and ActRNA$_n^{Ala}$ was carried out at 37˚C with (a) TuAlaRS (100 nM) and (b) EcAlaRS (100 nM).

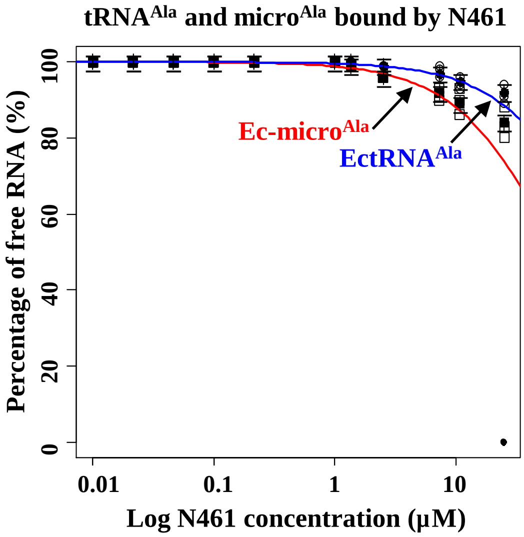

Ligand	K_d (μM)
Ec-micro^{Ala}	> 32
EctRNA^{Ala}	> 32

Supplementary Figure 4. Binding of EctRNA^{Ala} and Ec-micro^{Ala} by N461. The binding affinities of EcAlaRS-N461 for EctRNA^{Ala} and Ec-micro^{Ala} transcripts were determined by an EMSA with protein concentrations ranging from 32 to 0.0625 μM.

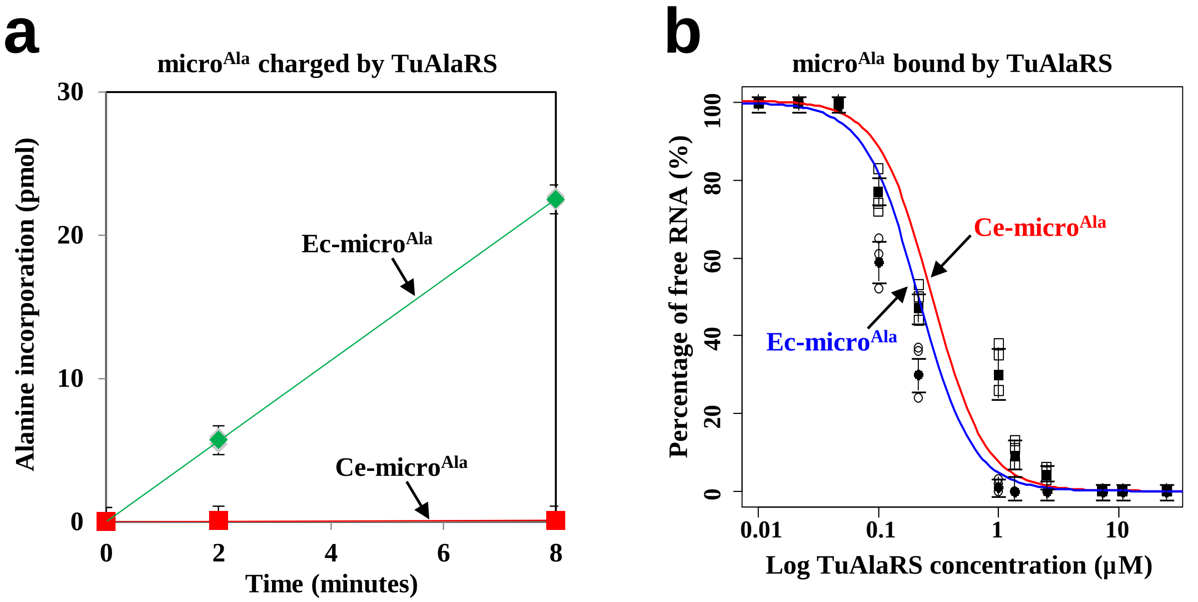

Ligand	K_d (μM)
Ec-micro^{Ala}	0.3 ± 0.1
Ce-micro^{Ala}	0.4 ± 0.1

Supplementary Figure 5. Aminoacylation and binding of Ec-micro^{Ala} and Ce-micro^{Ala} by TuAlaRS. (a) Aminoacylation of micro^{Ala} transcripts (5 μM) was carried out at 37˚C with 100 nM TuAlaRS. (b) The binding affinities of TuAlaRS for Ec-micro^{Ala} and Ce-micro^{Ala} transcripts were determined by an EMSA with protein concentrations ranging from 32 to 0.0625 μM.

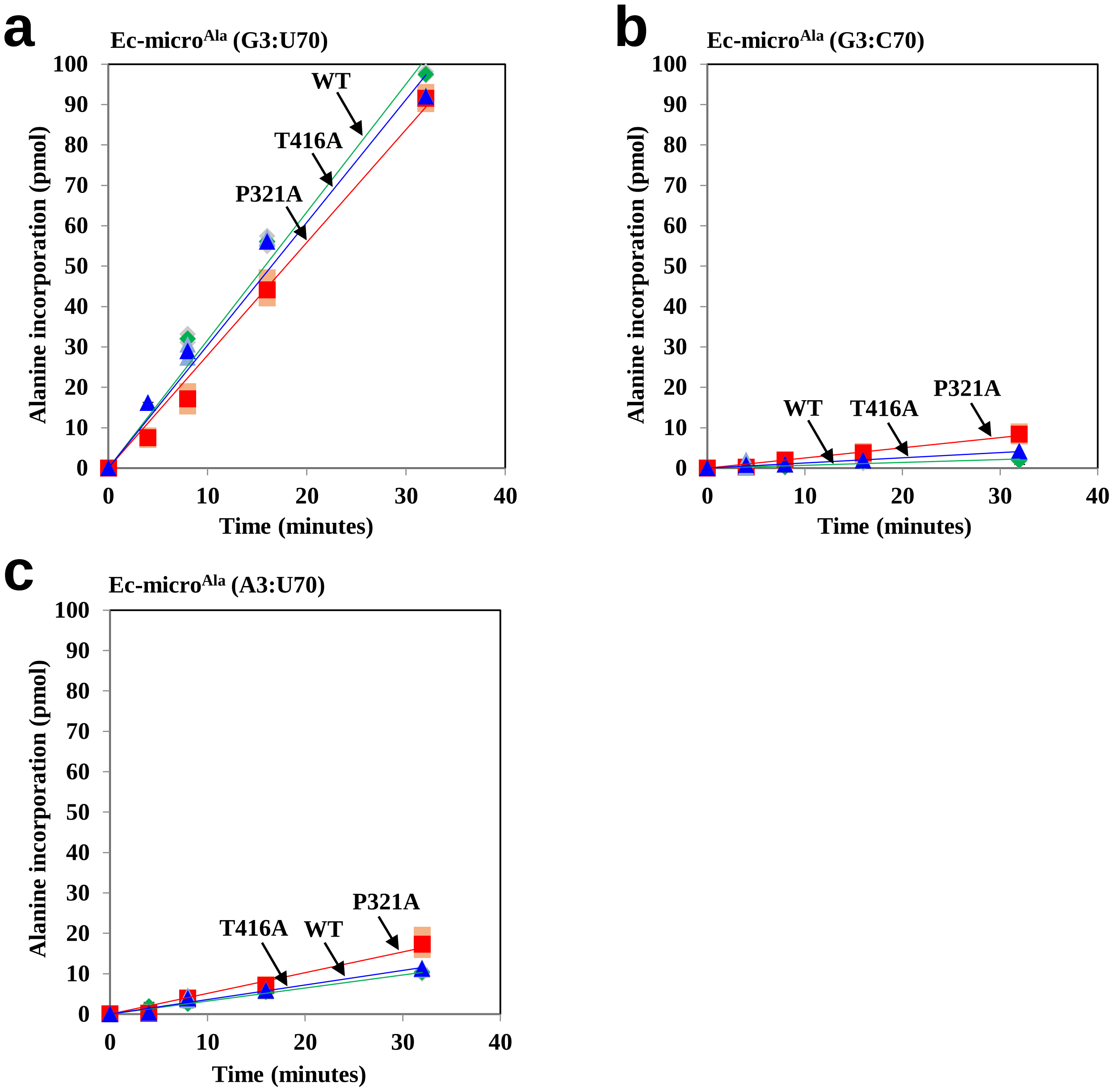

Supplementary Figure 6. Aminoacylation of Ec-micro^{Ala} by WT and mutant TuAlaRS enzymes. Aminoacylation of (a) Ec-micro^{Ala} (G3:U70) by 100 nM TuAlaRS enzymes, (b) Ec-micro^{Ala} (G3:C70) by 3 μM enzymes, and (c) Ec-micro^{Ala} (A3:U70) by 3 μM enzymes. The reactions were quenched after 4, 8, 16, and 32 minutes of incubation at 37℃.

Construct	Gene	Cloning vector	Rescue activity
① pTEF1	None	pTEF1	-
② pTT2	ScAlaRS	pTEF1	+
③ pTT121	TuAlaRS	pTEF1	+
④ pDEA4	EcAlaRS	pTEF1	+

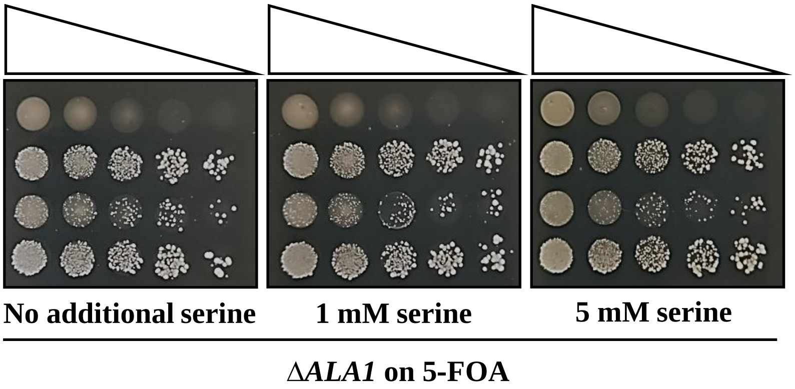

Supplementary Figure 7. Complementation assay of TuAlaRS on 5-FOA with additional serine. TuAlaRS's rescue activity was determined by transforming the test plasmid into a yeast *ALA1* KO strain and plating the resultant transformants on 5-FOA without and with additional serine (1 mM and 5 mM). The symbols "+" and "-" denote positive and negative complementation, respectively.

Supplementary Figure 8

Unedited/uncropped Western blot gels

Source data of Fig. 4

a

	Construct	Gene	Protein size (kDa)
①	pTEF1	None	-
②	pTT2	ScAlaRS	107
③	pDEA4	EcAlaRS	96
④	pTT126	EcAlaRS-N461	50
⑤	pTT121	TuAlaRS	47

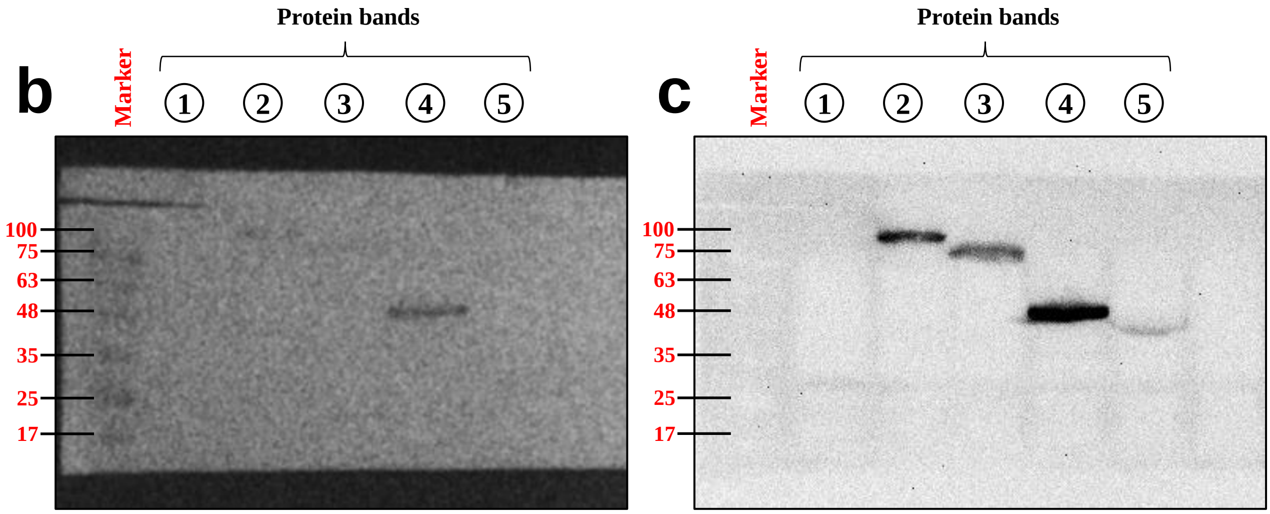

Supplement: Supplementary file 2 — Supplementary Information [file 42003_2023_4699_MOESM2_ESM.pdf]
