# Supplementary material for: A naturally occurring mini-alanyl-tRNA synthetase
Source: Commun Biol. 2023 Mar 23;6:314. doi: 10.1038/s42003-023-04699-0 (PMC10036535; doi:10.1038/s42003-023-04699-0)
Supplement: Supplementary file 3 — Description of Additional Supplementary Files [file 42003_2023_4699_MOESM3_ESM.pdf]

Description of Additional Supplementary Files

File name: Supplementary Data 1
Description: The numerical source data behind the graphs in the main figures.
